# Intercellular Communication in the Brain through Tunneling Nanotubes

**DOI:** 10.3390/cancers14051207

**Published:** 2022-02-25

**Authors:** Khattar E. Khattar, Janice Safi, Anne-Marie Rodriguez, Marie-Luce Vignais

**Affiliations:** 1 Institut de Génomique Fonctionnelle, University Montpellier, CNRS, INSERM, Montpellier, France; khattar.khattar@igf.cnrs.fr (K.E.K.); janice.safi@igf.cnrs.fr (J.S.); 2 University Paris Est Créteil, INSERM, IMRB, Créteil, France; anne-marie.rodriguez@inserm.fr

**Keywords:** tunneling nanotubes, mitochondria, brain, glioma, glioblastoma stem cells (GSCs), astrocytes, neurons, mesenchymal stem cells (MSCs), tumor microenvironment, degenerative brain diseases

## Abstract

**Simple Summary:**

Tunneling nanotubes (TNTs) are a means of cell communication which have been recently discovered. They allow the intercellular trafficking of many types of cellular compounds ranging from ions, such as Ca^2+^, to whole organelles such as mitochondria. TNTs are found in many tissues, both in physiological and pathological conditions. They are also found in the brain where they contribute to brain development and function and also to degenerative diseases and glioma.

**Abstract:**

Intercellular communication is essential for tissue homeostasis and function. Understanding how cells interact with each other is paramount, as crosstalk between cells is often dysregulated in diseases and can contribute to their progression. Cells communicate with each other through several modalities, including paracrine secretion and specialized structures ensuring physical contact between them. Among these intercellular specialized structures, tunneling nanotubes (TNTs) are now recognized as a means of cell-to-cell communication through the exchange of cellular cargo, controlled by a variety of biological triggers, as described here. Intercellular communication is fundamental to brain function. It allows the dialogue between the many cells, including neurons, astrocytes, oligodendrocytes, glial cells, microglia, necessary for the proper development and function of the brain. We highlight here the role of TNTs in connecting these cells, for the physiological functioning of the brain and in pathologies such as stroke, neurodegenerative diseases, and gliomas. Understanding these processes could pave the way for future therapies.

## 1. Introduction

The first reports of intercellular connections through tunneling nanotubes (TNTs) date back to 2004 [1,2]. At the time, the description of these thin structures, observed mainly in vitro, provoked great skepticism from the scientific community. This skepticism did not fade, even though similar findings were generated by laboratories around the world. It took 13 years before the biological consequences of the occurrence of these tunneling nanotubes were finally fully acknowledged [3]. TNTs changed the paradigm of a cell which could no longer be defined as delimited by its own plasma membrane, as TNT-connected cells actually share their plasma membrane. In addition, components of their cytoplasms from the smallest (e.g., calcium ions) to the largest (e.g., mitochondrial organelles) were no longer to be considered their very own but mobile entities since they were recognized as exchangeable between TNT-connected cells. This led to the concept that cells can form dynamic transient networks allowing the exchange of information (through the exchange of specific cellular cargoes) which ultimately allows these cells to efficiently respond to cellular challenges such as tissue repair following injury or response to cancer therapy. 

From a holistic standpoint, TNTs are expected to be the basis for constantly evolving cellular networks, linking together many different cell types and allowing exchanges of very diverse cellular cargoes, and thereby giving rise to dizzying combinations of intercellular regulations. Reviews on the general features of these tunneling nanotubes, their structural and functional characterization, as well as the biological effects of the transported cargoes, are numerous and instructive [4,5,6,7,8,9,10,11,12,13,14,15,16], and we refer to them for further information.

The subject of this review concerns more specific points, which remain key questions in the field. The first is what is the current evidence that TNTs indeed occur in vivo and in tissues. This is obviously a critical consideration whose resolution has been hampered by technical challenges [17]. Fortunately, the development of organoids and the improvement of imaging techniques have allowed progress in this field, as detailed below. A second key point concerns the signals that stimulate TNT formation and intercellular cargo trafficking. As described below, these signals can consist of both intracellular signaling cascades and extracellular signaling triggers. Deciphering these signals is essential for understanding the mechanisms of TNT-dependent intercellular cargo exchange. It will also provide clues for designing tools, either to enhance cargo exchange when beneficial to the recipient cells as for tissue repair, or conversely to inhibit the process when it is detrimental as when supporting resistance to cancer therapy.

The brain is an organ where cellular communication is essential for the many tasks it governs. Many different means for this communication have been described. TNTs are now found to contribute to an additional layer of this intercellular communication as they have been reported to connect together various cells of the brain, including neurons, astrocytes, microglia and pericytes, all of which participate in brain physiology. Besides, TNTs appear instrumental for degenerative pathologies such as Alzheimer’s disease (AD), Creutzfeldt-Jakob disease (CJD), Huntington’s disease (HD) and Parkinson’s disease (PD) (for reviews, refer to [18,19,20]. Brain cancer, in particular glioblastoma (GBM), is also a deadly disease. There too, TNTs promote disease progression, by allowing the microenvironment to support cancer cell proliferation and survival in response to treatment, as described below. It is expected and deeply wished that our growing knowledge of the biology inherent to these TNTs and to their transported cargoes will open up new avenues for cutting-edge research and for revolutionary therapeutic treatments for patients. 

## 2. Intercellular Communication through Tunneling Nanotubes (TNTs): A New Paradigm for Dynamic Cellular Interactions and Intercellular Exchange of Cellular Components

The formation of tunneling nanotubes (TNTs) has now been observed in numerous cell types in vitro and in diverse biological conditions (i.e., physiology, tissue damage, and cancer). These TNTs are thin tubes (less than a micron in width) that connect cells together and allow the transport of various cargoes, ranging from small molecules such as ions, of which Ca^2+^ has been the most studied, to microRNAs, protein aggregates responsible for neuro-degenerative diseases, to whole organelles such as endoplasmic reticulum and mitochondria. These cargoes can be exchanged between cells due to the very specific nature of these TNTs that act as “open conduits” between the connected cells. 

TNTs are the first intercellular nanotube structures described. Additional structures have since been described. Elegant cryoelectromicroscopy imaging showed that these individual TNTs can bundle up together, notably via N-cadherin binding, allowing the formation of tubular structures composed of multiple parallel TNTs [5,21]. Noteworthily, TNTs all appear to contain actin microfilaments which are determinant for TNT elongation and cargo trafficking [9]. 

Thicker cell-connecting tubes found in particular in tumors and called tumor-microtubes (TM) share many features of TNTs. In addition to actin microfilaments, they also contain microtubules which further support intercellular cargo transport and may contribute to the apparently longer lifespan of TMs compared to TNTs [22,23,24,25,26,27,28,29]. TMs and TNTs are not mutually exclusive as they were found to coexist in both glioblastoma organoids and 2D monolayer cultures [27,30]. This undoubtedly raises the question of what triggers one structure over the other and what are the biological consequences of the presence of either TNTs or TMs in tissues.

## 3. TNTs and TNT-Mediated Intercellular Cargo Trafficking Occur in Tissues

Even though TNTs have now been observed in many in vitro cell culture systems, the key question of their actual occurrence in tissues partly remains unclear. Demonstration of this presence was delayed by technical imaging challenges in the absence of specific TNT biomarkers. In addition to observing TNT- and TM-like cellular structures in resected tissues, such as tumors, observing tissues that are easily accessible due to their transparency, such as the retina, and tissue substitute systems such as organoids provide a more accurate picture of the extent of TNT/TM presence in tissue.

### 3.1. TNTs in Resected Tissues 

Confocal microscopy was used to search for the presence of nanotube-like structures in surgical specimens. Such TNT structures were reported in whole-mount corneas of GFP chimeric mice between eGFP-expressing MHC class II^+^ immune cells [31]. TNTs were also reported in human pleural mesothelioma and lung adenocarcinoma tumor specimens, with the caveat that these structures were visualized by the unexpected incorporation of Hoechst [32]. The use of fluorescent MitoTracker also enabled the visualization of TNTs in resected tissues from pancreatic ductal adenocarcinoma [33].

### 3.2. Organoids as a Tissue Surrogate to Demonstrate the Existence of TNTs In Vivo

Organoids are multicellular 3D-structures which aim at reproducing in vitro the anatomical structure of tissues with the goal of mimicking their in vivo functions. Tumor organoids are expected to allow to reproduce the tumor heterogeneity observed in vivo. Using such tumor organoids, the formation of both TNTs and TMs was observed among glioblastoma stem cells isolated from patients [27]. The formation of TNTs was also shown between human astrocytes and GBM cells in hyaluronic acid-gelatin based hydrogel 3D in vitro co-culture models [34]. Human cerebral organoid glioma (termed GLICO) were also developed from human embryonic stem cells and patient-derived glioma stem cells and shown to allow the formation of TMs which promoted the invasion capacity of the glioma cells [24].

Human cerebral organoids, also called mini-brains, can also be engineered through inkjet-bioprinting of cells, allowing the development of ex vivo structures that approximate the organization of the human brain in vivo, including the role of the neurovasculature [35]. These mini-brains have, as a goal, to recapitulate the physiological cell-cell and cell-matrix interactions which take place in a healthy brain and, beyond, to support the study of pathological disorders such as brain cancer and, besides, degenerative diseases such as Alzheimer’s disease and Parkinson’s disease and their responsiveness to therapy [35,36]. Such mini-brains were used to study the interactions between glioblastoma cells and other cells found in the brain, such as macrophages and endothelial cells [36,37]. Even though 3D-bioprinting techniques still need adjustments to get mini-brains closer to the cyto-architecture of the brain, 3D imaging improvements [38] could make mini-brains a valuable experimental system, in the near future, to detect TNT formation in GBM, determine how they are regulated by therapy and, ultimately, how they can influence the glioma cell response to therapy.

### 3.3. Murine In Vivo Models for the Observation of TNT Formation and Cargo Transfer

#### 3.3.1. Murine Models of Intercellular Mitochondria Transfer

The first evidence that mitochondrial transfer occurs in vitro was provided by landmark studies that documented mitochondrial transfer from MSCs to damaged bronchial epithelial cells in two murine models of acute lung injury triggered by rotenone and lipopolysaccharide (LPS), respectively [39,40]. Similarly, injection of B16ρ° mouse melanoma cells into syngeneic C57BL/6Nsu9-DsRed2 mice, which produce fluorescently labeled mitochondria, demonstrated in vivo transfer of mitochondria into these cells [41]. However, although these studies failed to properly provide information on the in vivo presence of TNTs, they have greatly advanced the field by documenting the in vivo occurrence of mitochondrial transfers.

#### 3.3.2. Murine Organs: Retina and Cornea

A number of observations of the in vivo occurrence of TNTs were made in mice retina and cornea. This can be explained by the transparency of the ocular media which makes it accessible by optical in vivo imaging [42]. The retina is of particular interest since it constitutes by itself a structure of the central nervous system. TNTs could thus be observed between pericytes of the retina in NG2–DsRed murine models which express the red fluorescent protein (DsRed) under control of the NG2 promoter, thus allowing the selective visualization of retinal pericytes [43]. These actin-containing interpericyte TNTs (IP-TNTs), 0.5 μm in diameter on average and up to 90 μm in length, contained both mitochondria and calcium ions. Interestingly, intercellular Ca^2+^ waves could propagate between the IP-TNT-connected pericytes and were essential for neurovascular coupling [43]. 

The formation of nanotubes connecting photoreceptor neurons (^Ph^NTs) was also observed in the mouse retina between transplanted and endogenous photoreceptors where they allowed the exchange of intracellular material, including proteins, mRNA and mitochondria in vivo [44]. Likewise, photoreceptors were shown to form NT-like structures in vitro, as either thin actin-containing NTs or thicker NTs (wider than 0.7 um) which also contained tubulin, allowing intercellular transfer of lysosomes and mitochondria [45]. Following transplantation of Nrl.Gfp^+/+^ donor photoreceptors, these ^Ph^NTs were also observed in vivo between these GFP donor cells and the photoreceptors of the recipient eyes. Importantly, the use of chimeric mice showed that ^Ph^NT-dependent process occurred both in the intact and damaged retina, suggesting a role of these ^Ph^NTs in both transplanted retinas and in retinal physiology [45].

#### 3.3.3. Murine Cerebral Cortex

TNTs were also detected between astrocytes and neurons in the cerebral cortex of mice expressing EGFP in astrocytes (AAV-*GFAP*-EGFP-p2A-cre murine models). By day 10 following viral injection, EGFP was found to transfer, through the F-actin-containing TNTs, from the EGFP-expressing astrocytes to the connected neurons [46].

#### 3.3.4. Xenografts of Human Tissues: Brain Tumors

When tissue transparency is an issue, as for the brain, alternative experimental methods were adapted which consisted in implanting a cranial glass window following the opening of the mouse skull and transplanting of the patient-derived glioblastoma cells. Glioblastoma cells expressing the GFP fluorescent protein could be monitored and their connections through tumor microtubes detected by longitudinal intravital two-photon microscopy following their implantation into the mouse brain [22,25,29,47]. TNTs, or at least one consequence of their occurrence such as intercellular mitochondria transfer, were shown in xenografts of other cancer cell types such as acute myeloid leukemia (AML) [48].

## 4. Molecular Determinants of TNT Formation and Cargo Intercellular Exchange

A number of studies have been carried out with the goal of identifying the molecular mechanisms for TNT formation and cargo transport. This has led to the identification of a series of proteins, as detailed below and reported in Table 1. Moreover, triggers for these processes were also identified (see below and Table 2). Interestingly, some triggers of TNT formation were found to be effective in some cell types and not in others, suggesting cell type specificity in these processes (see also [9]). Establishing whether the currently identified mechanisms for TNT formation and cargo exchange will be general or specific to given cell types will likely require further work.

### 4.1. Cell Components and Signaling Pathways Involved in TNT Formation and Cargo Trafficking 

#### 4.1.1. Proteins Linked to TNT Formation by Cytoskeletal Remodeling

The TNTs all appear to contain actin microfilaments, while the thicker ones also have a microtubule cytoskeleton [16], therefore warranting studies on the role of proteins involved in cytoskeletal remodeling in the process of TNT formation [49,50]. From this perspective, the cytosolic protein M-Sec, also known as B94, was shown to interact with Ral GTPase and the exocyst complex involved in the cytoskeleton actin remodeling. Knocking down either M-Sec or Ral GTPase suppressed TNT formation and reduced the propagation of calcium flux between Raw264.7 murine macrophages [50]. The M-Sec-dependent TNT formation was shown to require its interaction with the endoplasmic reticulum chaperone protein ERp29, as demonstrated in the human osteosarcoma U2OS cell line [51]. Moreover, the transmembrane MHC class III protein leukocyte specific transcript 1 (LST-1) was also identified for its role in assembling different proteins, including M-Sec and RalA, involved in tunneling nanotube formation [52]. RalGPS2 is a Ras-independent guanine nucleotide exchange factor for the RalA GTPase. It was demonstrated to allow TNT formation in bladder and kidney cancer cells through its interaction with Akt and PDK1, leading to activation of the Akt/PI3K/mTOR pathway, as well as with LST-1 and RalA [53]. The exocyst complex has been further involved in TNT-mediated cargo trafficking as Sec3 and Sec5 proteins, which are part of this complex, were identified at the site of actin cytoskeleton recruitment in TNTs between natural killer cells and MDA-MB-231 breast cancer cells and shown to promote mitochondria trafficking between these cells [54]. In addition to the RalA GTPase, the two Rho GTPases Rac1 and Cdc-42 have been involved in TNT formation, in RAW/LR5 macrophages, by allowing the activation of their downstream effectors WAVE2 and WASP, respectively [49]. Eps8 (Epidermal growth factor receptor pathway 8) is another factor interacting with PI3K. Eps8 regulates actin remodeling. The role of Eps8 for TNT formation was shown in PC3 and LNCaP prostate cancer cells [55]. More precisely, its actin filament bundling activity was shown essential for TNT induction in neuronal CAD cells [56]. Finally, the actin-based molecular motor myosin-X (Myo10) was also demonstrated to favor TNT formation between neuronal CAD cells [57].

#### 4.1.2. Signaling Pathways Involved in TNT Formation

Under oxidative stress, triggered by hydrogen peroxide (H_2_O_2_) or serum depletion, p53 activation and the consequent upregulation of EGFR expression and Akt/PI3K (phosphoinositide 3-kinase)/mTOR (mammalian target of rapamycin) activation were found to promote the establishment of TNTs among astrocytes, possibly through M-Sec and the exocyst complex [58]. In murine CAD neuronal cells, the Wnt/Ca^2+^ pathway was shown to contribute to both TNT formation and cargo trafficking through TNTs [59]. Activation of Ca^2+^/calmodulin-dependent protein kinase II (βCAMKII) by the Wnt/Ca^2+^ pathway was proposed to support TNT formation through a two-step mechanism, involving Wnt/Ca^2+^-dependent detachment and subsequent reattachment of βCAMKII to the actin cytoskeleton [59]. Noteworthily, the Rab GTPase protein Rab35 and its downstream signaling through ACAP2 (Arf-GAP with coiled-coil, ankyrin repeat and PH domain 2), ARF6-GDP and EHD1 (EH domain-containing 1) also promoted TNT formation in CAD cells [60].

#### 4.1.3. Proteins Involved in Cargo Trafficking through TNTs 

Miro-1 (mitochondrial Rho GTPase-1) was found essential for the mitochondrial trafficking between mesenchymal stem cells (MSCs) and lung bronchial epithelial cells in a murine model of lung injury (Ahmad et al., 2014). Miro-1 expression in MDA-MB-231 breast cancer cells is important for the TNT-mediated acquisition of mitochondria from natural killer cells [54]. The role of the gap junction protein connexin 43 (Cx43) was shown in vivo for the connection of BMSCs to lung epithelial cells, in a murine model of lung damage, and for TM connections between astrocytoma cells, allowing mitochondria and calcium intercellular exchange, respectively [25,40] (see also [61,62] for review). Two neuronal proteins, the growth associated protein (GAP-43) and Tweety-homolog 1 (Tthy1), were also described as molecular drivers of TM formation and function, as shown in glioma in vivo models [22,25,47]. Finally, in multiple myeloma, the membrane protein CD38 was also reported to support TNT formation and mitochondrial transfer from bone marrow stromal cells, as shown in vitro and in vivo after implantation of CD38 KD cells in NSG mice [63].

### 4.2. Extracellular Triggers and Regulators of TNT Formation and Cellular Cargo Exchange

A key question about TNTs is what are the physiological and pathological conditions which regulate these intercellular communications and the cargo trafficking that ensues. It is now generally acknowledged that cellular and metabolic stress trigger TNT formation. Among the known ROS (reactive oxygen species), hydrogen peroxide (H_2_O_2_) constitutes an important trigger of TNT formation as shown for instance for rat astrocytes, murine neuronal CAD cells and cocultures of rat astrocytes and C6 glioma cells [57,58,64,65,66]. H_2_O_2_ also enhanced the formation of TNTs and mitochondrial transfer from human bone marrow stromal cells (BMSCs) to CD34^+^ cells from patients with acute myeloid leukemia (AML), an effect relying on the NADPH oxidase-2 (NOX2)-dependent production of ROS [67].

Other extracellular stimuli can support cellular metabolic stress. They include acidic microenvironment (pH 6.6), hypoxia (1% O_2_), and serum starvation [32,53,55]. These metabolic stress induced TNT formation in human PC3 and LNCaP prostate cancer cell lines via the stress-induced chaperones clusterin (CLU) and YB-1 (Y-box binding protein-1) and PI3K/Akt activation [55]. Similarly, serum depletion induced TNT formation in human malignant pleural mesothelioma [32]. Interestingly, these stimulatory triggers were shown to act in a cell type-specific manner, promoting TNT formation in some cell types but not in others. As an example, oxidative stress (H_2_O_2_) promoted TNT formation in HEK293 cells while it had no effect on 5637 bladder cancer cells. On the other hand, 5637 cells showed greater TNT formation under acidic conditions as well as under serum deprivation conditions [53]. Besides, as expected from the observed TNT stimulatory effect of PI3K/Akt/mTOR signaling, inhibition of mTOR activity, by rapamycin or by the rapamycin derivative everolimus, suppressed TNT formation in murine astrocytes and human mesothelioma MSTO-211H cells, respectively [32,58]. 

Tissue damage, localized at a drug injection site or to the larger area of tumor resection, was also identified as sufficient cellular stress to trigger TNT formation [31,47]. Moreover, cancer therapy itself can constitute a tissue insult sufficient to stimulate TNT formation, as shown following α-particle radiation of U87 glioblastoma cell line or doxorubicin, cytarabine and temozolomide chemotherapeutic treatments in human pancreatic cell lines, human AML blasts and human glioblastoma cell lines respectively [33,48,68,69].

Pathologies can also generate cellular modifications which support TNT formation. During viral infection with the human immunodeficiency virus HIV-1, the viral protein Nef, together with the cellular protein M-sec, were shown to stimulate TNT formation in monocytes derived macrophages (MDMs) [70]. Likewise, metapneumovirus (HMPV) infection of human bronchial epithelial BEAS-2B cells generated an increased number and longer TNTs mediated by the HMPV P phosphoprotein [71]. The alphaherpesvirus US3 protein kinase also induced the formation of stable TNTs, allowing the transport of pseudorabies virions and allowing alphaherpesvirus spread [72]. 

In the development of neurodegenerative diseases, infectious prion PrP^Sc^, α-synuclein, mutant Huntingtin (mHTT) and Tau aggregates have been shown to increase TNT formation and consequently facilitate the intercellular transfer of the toxic protein aggregates, leading to the spreading of the disease to other cells [73,74,75,76,77]. It should be noted that, in addition to the misfolded protein aggregates they generate that promote TNT formation, cells with neurodegenerative diseases also produce H_2_O_2_ which, as discussed above, can further contribute to TNT formation [78,79].

## 5. Focus on an Organ: The Brain Role of Intercellular Communication in Physiology and Pathology

The brain contains many different cell types including neurons, astrocytes, oligodendrocytes, microglia, and endothelial cells. Intercellular communication between these different cell populations is essential for maintaining the homeostasis of the central nervous system (CNS) [80,81,82]. Communication between these cells is mediated by various means, which can rely on diffusible paracrine factors, such as neurotrophic factors and neuropeptides or on extracellular vesicles (EVs). Communication between brain cells may also rely on voltage-gated channels, active membrane transporters, and diffusion channels, as thoroughly reviewed elsewhere [83]. Furthermore, intercellular communication can rely on the close proximity of interacting cells, as for chemical and electrical synapses, or require physical contacts as it is the case for gap junctions and TNTs, as detailed below.

### 5.1. Cell Communication through Paracrine Secretion in the Healthy Brain

#### 5.1.1. Communication through Soluble Factors

A flurry of studies have provided evidence that soluble factors secreted by neural, glial and microglial cells as well as by cells of the blood-brain barrier, such as endothelial cells end pericytes, play a prominent role in the regulation of neurogenesis and gliogenesis processes [84]. Astrocytes, the most abundant cells in the CNS, are also considered the most potent secretory glial cells capable, through their secretome, of controlling the behavior and activities of neurons, oligodendrocytes, microglia, endothelial cells and pericytes. This secretome includes ciliary neurotrophic factor (CNTF), bone morphogenetic protein (BMP), brain-derived neurotrophic factor (BDNF), fibroblast growth factor 2 (FGF2), nerve growth factor (NGF), platelet derived growth factor-AA (PDGF-AA), vascular endothelial growth factor (VEGF), transforming growth factor-β (TGF-β), glial cell line-derived neurotrophic factor (GDNF), tumor necrosis factor-α (TNFα), IL-6, IL-1β, IL-10, and IL-33 [85,86,87,88,89]. 

In addition to astrocytes, many other cells of the brain communicate through their paracrine activity [87,90], as evidenced by: 

(i) the crosstalk between neurons and CNS stem cells via the secretion by neurons of BDNF, neurotrophin-3 and BMP, which leads to the neuronal differentiation of CNS stem cells [84]. Neurons also promote the proliferation/differentiation of oligodendrocytes and Schwann cells via their secretion of neuroregulins, such as glial growth factor 2 (GGF2) [91], 

(ii) the BDNF-dependent modulation by oligodendrocytes of synapse activity in neurons [92]

(iii) the regulation of astrocyte differentiation by endothelial cells, through leukemia inhibitory factor (LIF) release [93], and finally,

(iv) the role of microglia in promoting neuroprotection and neurogenesis through the production of BDNF [94], IGF-1 [95], TGF-β [96] and arginase 1 [97]. 

Other factors, in addition to growth factors and cytokines, allow brain cells to communicate with each other. For instance, neurons and astrocytes communicate with surrounding cells through the secretion of (i) neurotransmitters such as glutamate, ATP, and GABA; (ii) neurotransmitter precursors such as glutamine and proenkephalin (PENK); (iii) neuromodulators such as D-serine; (iv) peptides and hormones such as atrial natriuretic peptide and thyroid hormones; (v) eicosanoids and metabolic substrates such as lactate, citrate and glucose; and (vi) ROS scavengers including glutathione and ascorbate (for review, see [98]).

#### 5.1.2. Communication through Extracellular Vesicles

Brain cells can interact with each other through the secretion and internalization of extracellular vesicles (EVs) including exosomes and microvesicles. In the brain, EVs were shown to carry diverse cargoes including growth factors and cytokines, signaling proteins, lipids as well as genetic material including mRNA, miRNA, long non-coding RNA (lncRNA), and mtDNA [99]. This diversity can explain why EV-mediated crosstalk exerts a wide range of biological effects. For instance, microglia-derived EVs containing inflammatory cytokines and GAPDH trigger the propagation of inflammatory signaling throughout the brain [100] while EVs carrying endocannabinoid N-arachidonoylethanolamine (AEA) on their surface inhibit the synaptic transmission between neurons by abrogating GABA release [101] and enhancing their sphingolipid metabolism [102]. As another example, EVs produced by astrocytes containing Apolipoprotein D or miR-92b-3p have been found to exert neuroprotective and neuroregenerative effects after exposure to ATP, IL-10 or ischemic insult [103,104]. Finally, the release of miR-124-3p-containing exosomes from neurons has been reported to decrease inflammation in the brain, by suppressing activation of neuro-inflammatory M1 microglia and A1 astrocytes [105].

### 5.2. Cell Communication through Gap Junctions 

Cell communication can also occur through direct physical interactions, as it is the case for gap junctions. Gap junctions (GJs) are intercellular channels composed of connexins. These structures allow the diffusion of small molecules including ions, neurotransmitters, second messengers and metabolites between connected cells. GJ-mediated communications are involved in the transmission and propagation of neuronal signals, potassium clearance at the synaptic level and calcium signaling [106]. For instance, astrocytes that abundantly express connexin 43 (Cx43) communicate through (i) Cx43/Cx43 GJs with other astrocytes for the synchronization of their activities [107]; (ii) through Cx43/Cx47 GJs with oligodendrocytes to promote myelination [108]; and (iii) through Cx43/Cx36 GJs with neurons to improve their survival [107]. In addition, Cx43 is abundantly expressed at astrocytic endfeet that contact blood vessels, where it plays a critical role in maintaining the integrity of the brain blood barrier [109]. Gap junctions are also important components of electrical synapses as they allow the passive flow of electrical currents between pre-and post-synaptic neurons, resulting in the generation of post-synaptic action potential [110].

### 5.3. Cell Communication through TNTs

Several in vitro and in vivo studies have reported that brain cells communicate through intercellular-TNT mediated connections. Based on the ability of TNTs to mediate the transfer of a broad range of cellular compounds including ions, proteins, vesicles, genetic material and of organelles such as lysosomes or mitochondria, these structures have been suggested to be implied in neonatal brain development and adult brain homeostasis [16]. In particular, several in vitro and in vivo studies have reported the TNT-mediated connection between astrocytes and neurons in neonatal and adult brain [111,112,113]. 

In the neonatal brain, TNT-mediated crosstalk has been shown to mediate electrical coupling and calcium wave propagations between astrocytes and immature neurons [113]. Since calcium signaling was reported to be involved in the proliferation, migration and differentiation of immature neurons [114], these observations suggest that astrocytes contribute to neuronal maturation through TNTs. This hypothesis was further strengthened by studies revealing the presence of TNT structures linking astrocytes and neurons, as observed in mouse neonatal brain slices [111,112]. In addition, TNT connections have been observed amongst pericytes and between pericytes and endothelial cells in human fetal brain, suggesting that these structures may play a role in the early phases of brain vascularization [115].

In the adult brain, the crosstalk of astrocytes and neurons by the means of TNTs has been reported in vivo, although the exact contribution of this phenomenon to the maintenance of homeostasis in the healthy adult brain remains unclear [46]. Still, in vivo studies suggest that TNTs are involved in the regulation of brain function. For instance, TNTs have been involved in the regulation of neurovascular coupling and in the adaptation of blood flow to neuronal activity by allowing the transfer of calcium ions between interconnected pericytes [43].

Another function of TNTs is their ability to detoxify the healthy brain from toxic cellular compounds such as misfolded proteins that can accumulate in neurons but also in astrocytes and microglia [116]. In particular, TNTs have been found in vitro to convey fibrillar α-synuclein (α-syn) cargoes from α-syn –overloaded astrocytes to microglia where it is degraded. Similarly, it was recently reported that α-syn-overloaded microglia have the capacity to form TNTs, as observed both in vitro and in vivo, allowing them to discharge their excessive α-syn aggregate contents to neighboring naive microglia, which take care of their clearance [117]. Beyond their role in α-syn degradation, the TNTs that connect naive microglia to affected microglia have also been shown to mediate the transfer of mitochondria to these affected microglia, thereby preventing cell death and inflammation [117]. Finally, TNT-mediated transfer of misfolded protein aggregates between neurons has been documented in vitro in several studies [59,74,75]. Interestingly, protein assemblies were transferred along TNTs inside lysosomes, presumably for their degradation by neurons initially devoid of these toxic compounds [74]. 

### 5.4. Stroke—Role of TNTs in Regeneration

The formation of TNTs can be increased in the brain under stress conditions, including ischemic and oxidative insults [64]. Acute ischemic damage causes dramatic mitochondrial dysfunction in neurons, jeopardizing their viability, and TNT-mediated mitochondrial transfer appears to be an adaptive mechanism for maintaining mitochondrial function in the damaged brain and promoting recovery after stroke [118]. Excess ROS production by damaged cells has been shown to trigger TNT formation, notably in hydrogen peroxide-injured astrocytes [66,119], leading to mitochondria transfer from the healthy to the damaged cells [12,64,120].

Most brain cells, including astrocytes, neurons, microglia, and endothelial cells, have been reported to respond to stroke or stroke-mimicking damage conditions by TNT formation and mitochondria transfer, in a pro-survival mechanism. This was shown in vitro for the transfer of mitochondria from healthy neurons and astrocytes to stressed neurons, supporting their rescue [121,122,123]. By a similar mechanism relying on TNT-mediated mitochondria transfer, neural stem cells could protect apoptotic brain microvascular endothelial cells against cell death, as shown in vitro and in vivo [124]. 

In addition to transporting healthy mitochondria to damaged cells, TNTs also participate in neuro-regenerative processes by conveying cargoes, such as dysfunctional cellular compounds or stress signals, thereby eliciting an adaptive repair response in recipient cells. As an example, neurons were shown to release dysfunctional mitochondria to astrocytes, leading to their degradation and disposal [125]. It is worth noting that, outside of the brain, the transfer of dysfunctional mitochondria from damaged cells to healthy cells, such as MSCs, has also been shown to be sensed as a stress signal leading to the activation of tissue-repair processes [120,126]. Accordingly, a similar TNT-mediated phenomenon could occur in the ischemic brain where damaged nerve cells could transfer dysfunctional mitochondria to healthy cells (including nervous stem cells), thereby triggering a regenerative response to brain injury.

Brain ischemia was also reported to increase TNT communication between microglia and neurons, a process that may confer neuroprotection by ensuring microglia removal of neuronal debris and regulating inflammation [127,128]. Finally, mast cells were also shown to communicate with each other in vitro, through TNT-mediated transfer of secretory granules and mitochondria [129]. Mast cells are long-lived cells and major effectors of allergic inflammation which are also found in the brain [130,131]. It was hypothesized that mast cells could thus rapidly spread stress signals to neighboring cells to alert them of harmful conditions [129]. 

Beside their positive effects in stroke recovery, TNT communications may also be detrimental to recipient cells, as suggested by Bittins and Wang [132]. They showed that UV-treated apoptotic neurons transferred through TNTs pro-phagocytic signals, including phosphatidylserine, oxidized phospholipids and calreticulin, to cocultured viable neurons, leading to their phagocytosis by macrophages [132]. Finally, damaged brain cells, including neurons and astrocytes, were reported to communicate with each other through TNTs. Whether this phenomenon is beneficial or detrimental in the context of ischemic brain repair is not yet known [66,133,134]. 

### 5.5. Degenerative Brain Diseases

TNTs allow the spreading and progression of neuronal diseases such as Alzheimer’s disease (AD), Creutzfeldt-Jakob disease (CJD), Huntington’s disease (HD) or Parkinson’s disease (PD) through the intercellular dissemination of their respective pathogenic agents: Tau and amyloid-β aggregates for AD [73,76,135], PrP^Sc^ prions for CJD [136], mutant huntingtin aggregates for HD [75,137], and α-synuclein (α-syn) aggregates for PD [74,116,138,139]. Interestingly, in addition to the dissemination of toxic protein aggregates, the intercellular exchange of mitochondria via TNTs were also proposed to promote the progression of Parkinson’s disease [140].

## 6. Brain Tumors

Among brain tumors, glioblastoma (GBM) stands out as the most devastating, with an associated bleak prognosis. This is often related to a delayed diagnosis of the tumor, to the point where it already spread to an important area of the brain and therefore impairs cognitive functions. Full GBM surgical resection can be made difficult by the infiltrative nature of these tumors and by the need to preserve essential cognitive functions of the patients. From a cellular standpoint, the difficulty in efficiently treating glioblastoma also stems from the high heterogeneity and plasticity of these tumors, beyond the still debated question of the cell of origin of these tumors [141,142,143,144,145,146]. 

The glioblastoma stem cells (GSCs) present in GBM widely contribute to GBM recurrence following surgery and to resistance to both radio- and chemotherapies [144,147,148]. These GSCs undergo highly dynamic changes of their genetic, epigenetic, and metabolic features, which supports their escape from GBM therapies [149]. Asymmetric cell division of GSCs also contributes to the generation of GSCs endowed with increased prosurvival features [150]. Moreover, these dynamic changes are supported by evolving interactions between GSCs and their microenvironment, via cytokines/chemokines, extracellular vesicles, and TNT/TM-mediated cargo exchange [23,149,151,152,153].

### 6.1. Biological Effects of Cell-Cell Connections among Glioblastoma Cells

As mentioned above, the Winkler laboratory has helped advance in the field by developing orthotopic xenograft models. Implantation of labeled glioblastoma stem-like cells into mice skulls, followed by longitudinal intravital two-photon laser scanning microscopy, allowed for the observation of tumor microtube formation in vivo as well as of their effects on glioblastoma progression [22,25,29,47]. TM connections between glioblastoma cells were found to induce stemness features in these cells, as shown by RNA-seq analysis and increased expression of the stemness marker Nestin. This was linked to an increased capacity of glioblastoma cells to reinitiate tumor growth [29]. TM-connected glioblastoma cells also demonstrated enhanced proliferation and invasion capacities [25]. Importantly irradiation of the tumors, 60 days after tumor implantation in the mice brains, showed higher survival of the TNT-connected glioma cells, demonstrating higher resistance to radiotherapy [22,25,29]. Likewise, temozolomide treatment of the glioma xenografts, at D85 following engraftment, demonstrated higher survival in the highly connected glioblastoma cells, indicating enhanced chemoresistance of these cells [47].

Human glioma organoids obtained from patient-derived glioma stem cells also constitute valuable experimental approaches for deciphering the role of TNTs in glioma progression. They were shown to allow the formation of both tunneling nanotubes and tumor microtubes [24,27]. Glioma cells connected by TMs demonstrated an enhanced capacity to invade the surrounding normal host tissues [24]. The use of glioblastoma stem cells expressing fluorescent mitochondria (MitoGFP) also showed mitochondria trafficking through the TNT-like structures. The percentage of mitochondria-receiving cells increased over the 23 days of glioblastoma organoid culture, demonstrating dynamic cellular interactions and mitochondrial exchange among GSCs [27].

TNTs among GBM cells were also shown to allow the transfer of the DNA repair enzyme O^6^-methylguanine-DNA methyltransferase (MGMT), from MGMT-expressing GBM cells to MGMT-defective GBM cells [69]. As the expression of MGMT by glioblastoma cells has been linked to resistance to the alkylating agent temozolomide, this TNT-mediated transfer of MGMT was proposed as spreading this MGMT-based resistance to TMZ among GBM cells.

### 6.2. Effect on Glioma Progression of TNT Connections between Glioma Cells and Other Cells of the Brain

The nervous system plays a major role in glioma initiation and progression, notably due to the interactions that neurons, astrocytes and glial cells can develop with glioma cells [154]. TNT-mediated connections were reported between rat astrocytes and glioma cells, where they were found to increase glioma cell proliferation [65]. Interestingly, human astrocytes were also observed to form TNTs with glioma cells, in 2D and 3D-cocultures which, in this case, resulted in increased GBM cell proliferation and resistance to chemicals including temozolomide, clomipramine and vincristine [34]. Conversely, TNT connections between human GBM cells and non-tumor astrocytes, and the resulting transfer of mitochondria from GBM cells to astrocytes, were shown to allow the tumor cells to modify the tumor microenvironment [155]. Interestingly, the TNTs formed between U87-MG cells (as a model of GBM cells) were found to be thicker than those formed between NHA astrocytes, raising the possibility of using these structures for therapeutic purposes, for instance with liposomes as drug delivery vehicle [156].

As a matter of fact, intercellular mitochondria transfers have been shown in many instances to support proliferation and resistance of cancer cells as well as repair of damaged cells (for review see [12,14]). In the brain, mitochondria transfer from astrocytes was shown to contribute to the rescue of cisplatin-treated neurons [157]. TNT-mediated mitochondria transfers have been observed between human mesenchymal stem cells (MSCs) and glioblastoma stem cells in vitro [8,14]. The transfer of MSC mitochondria to GSCs, by the experimental procedure of ‘Mitoception’ [158,159,160], allowed researches to demonstrate that MSC mitochondria enhance the energy metabolism of the GSCs in a dose-dependent manner, as well as their resistance to temozolomide treatment (Nakhle et al., unpublished results), thus further supporting the role of the glioblastoma microenvironment and of mitochondrial transfers in GBM progression.

## 7. Conclusion What’s Next in the Field?

In this field of intercellular connections through TNTs/TMs, which is now evolving at an accelerated pace, many experimental data were obtained recently, mainly due to novel techniques and experimental approaches, as mentioned in this review. Nonetheless, a number of questions still remain unanswered. Among these, the exact biophysical characteristics of these intercellular structures and the mechanisms involved in cargo transport still need to be determined. TNTs exist in different flavors, such as individual TNTs, bundles of individual TNTs, and thicker TNTs (also known as tumor microtubes). It is not known what triggers the formation of one of these structures rather than the others. Beyond the mere biophysical properties of these structures, expected to be distinct for each of them, addressing this question could become a priority if each type of these intercellular connections were to be more prone to convey specific cellular cargoes. This leads to the related question of how donor cells decide which organelles, molecules or signals they transfer through TNTs to the recipient cells and, conversely, whether recipient cells can request specific cargoes from their neighboring cells, to meet their needs.

TNTs have been shown to connect cells of either similar cell types, sometimes with different biological status, or alternatively cells of different cell types. This raises the question of the cell features which determine the direction of cargo trafficking between the TNT-connected cells. Is it just a matter of intracellular concentrations of the transported cargoes? Or is it rather the biological context, for instance physiological versus pathological? In the latter case, is there an influence of local inflammatory conditions with their payload of circulating cytokines? Alternatively, is it the metabolic state of the tissues, including the production of reactive oxygen species (ROS) as mentioned above, that determines the exact nature of the TNT-transported cargo? This would mean that the cellular and tissue microenvironment would play a major role on the exchanged cargoes and the resulting cellular functions. 

Finally, if we consider the cellular complexity of a tissue or organ, what percentage of these cells are connected through TNTs at any given time? Is the dizzying vision of a matrix of connections linking all cells and allowing real-time exchange of a diversity of cellular constituents a reality? As described above, tissue imaging allowed thorough progress and the establishment of the actual occurrence of these TNTs as well as the identification of some cellular cargoes in vivo. However, following the real-time trafficking of all possible cargoes in vivo, as well as their combined functional effects, is another challenge that will require further work. Future findings in this field will be valuable to determine the exact in vivo contribution of TNTs, both in maintaining tissue homeostasis in physiological conditions and in promoting tissue repair following injury. In addition, it will be of primary importance to assess to what extent TNTs are responsible for the initiation or aggravation of brain diseases, including cancer or neurodegenerative diseases such as Parkinson’s or Alzheimer’s syndromes. Therefore, for now, we can speculate that we are only seeing the tip of the iceberg regarding the extent and biological effects of these TNT-mediated cargo exchanges in tissues.

## Figures and Tables

**Table 1:** Proteins involved in TNT formation and cargo transport.

	Proteins	Connected Cells	Molecular Mechanism	Cargo Transport	References
**TNT formation by cytoskeletal remodeling**	**M-sec**	Raw264.7 macrophages	Interaction with Ral GTPase and exocyst complex for cytoskeleton actin remodeling	Ca^2+^	Hase et al., 2009
	**RalGPS2**	5637 bladder cancer cells, HEK293 kidney cancer cells	Interaction with Akt and PDK1 leading to activation of Akt/PI3K/mTOR signaling Interaction with RalA and LST1	Mitochondria RalA, LST1	D’Aloia et al., 2021
	**Sec3/Sec5**	Immune cells and breast cancer cells	Colocalization at the site of actin cytoskeletal recruitment	Mitochondria	Saha et al., 2022
	**ERp29**	Osteosarcoma U2OS cell line	Interaction with M-sec	Mitochondria HIV virus	Pergu et al., 2019
	**LST-1**	HeLa cells, HEK-293T cells	Assembly of M-sec and Ral GTPase at the plasma membrane leading to actin polymerization	DiI-labeled vesicles	Schiller et al., 2012
	**Rac-1, Cdc42**	RAW/LR5 macrophages	Activation of WASP & WAVE2 followed by Arp2/3 complex regulation of actin cytoskeleton	DiI labeled material	Hanna et al., 2017
	**Myo10**	CAD cells	Not defined	DiD-labeled vesicles	Gousset et al., 2013
	**Eps8**	CAD cells	Bundling actin filaments	DiD-labeled vesicles	Delage et al., 2016
		PC3 cells	Actin remodeling	CLU, YB-1	Kretschmer et al., 2019
**Signaling pathways**	**P53**	Astrocytes	Upregulation of EGFR and Akt/PI3K/mTOR, M-sec overexpression activation of Ral-exocyst complex	ER, Golgi endosomes mitochondria	Wang et al., 2011
	**Wnt/Ca^2+^**	CAD cells	Activation of βCAMKII actin polymerization/TNT stabilization	DiD-labeled vesicles, α-synuclein	Vargas et al., 2019
	**Rab35**	CAD cells	Activation of ACAP2/inactivation of ARF6 recruitment of EHD1 for TNT formation	DiD-labeled vesicles	Bhat et al., 2020
**Cargo trafficking through TNTs**	**Miro-1**	MSCs and bronchial epithelial cells	Not defined	Mitochondria	Ahmad et al., 2014
		Immune cells and breast cancer cells	Not defined	Mitochondria	Saha et al., 2022
	**Connexin43**	BMSCs and lung epithelial cells	Ca^2+^ dependent mechanism	Mitochondria Ca^2+^	Islam et al., 2012
		Astrocytoma cells	Not defined	---	Osswald et al., 2015
	**GAP43**	Neuronal cells	Not defined	Ca^2+^	Osswald et al., 2015; Weil et al., 2017
	**Tthy1**	Neuronal cells	Not defined	---	Jung et al., 2017
	**CD38**	BMSCs and multiple myeloma cell lines	Not defined	Mitochondria	Marlein et al., 2019

**Table 2:** Regulation of TNT formation and cargo transport.

Stimuli		Connected Cells	Molecular Mechanisms	Cargo Transport	References
**Cellular stress**	**ROS** **(H_2_O_2_)**	Neuronal CAD cells	Not defined	DiD-labeled vesicles	Gousset et al., 2013
		Astrocytes T lymphocytes adult mammalian stem cells to somatic cells	mROS cytochrome c caspase 3	Mitochondria, Fas ligand	Rustom, 2016
		Astrocytes, neurons	p53 activation	ER, mitochondria, Golgi, endosomes, intracellular and extracellular amyloid β, Alexa488 dye	Wang et al., 2011
		Astrocytes C6 glioma cells	p53 activation	Mitochondria	Zhang and Zhang, 2015
		Astrocytes	p38 MAPK activation	Colocalization of myosin with F-actin in the TNTs	Zhu et al., 2005
		AML cells, BMSC, nonmalignant CD34+	NOX2	Mitochondria	Marlein et al., 2017
		HEK 293 cells	RalGPS2-RalA pathway	---	D’Aloia et al., 2021
	**Acidic** **microenvironment**	Mesothelioma cells	mTOR pathway	Vesicles, proteins, mitochondria	Lou et al., 2012
		5637 bladder cancer cells	RalGPS2 upregulation	Mitochondria, RalA, LST1	D’Aloia et al., 2021
	**Serum deprivation**	Neurons astrocytes	Akt, PI3K and mTOR	Amyloid beta	Wang et al., 2011
		5637 bladder cancer cells	RalGPS2 upregulation	Mitochondria, RalA, LST1	D’Aloia et al., 2021
	**Tissue damage**	S24 and T269 glioma cell lines	---	---	Weil et al., 2017
**Cancer therapy**	**α-particle** **radiation**	Glioblastoma U87 cells	---	---	Matejka et al., 2020
	**Doxorubicin**	Pancreatic adenocarcinoma	---	Chemotherapeutic drug	Desir et al., 2018
	**Cytarabine**	AML, BMSCs	---	Mitochondria	Moschoi et al., 2016
	**Temozolomide**	Glioblastoma cells	---	Receptors (CCR5, CXCR4, LRP1) mitochondria Vesicles, MGMT	Valdebenito et al., 2020
**Viral infection**	**Nef (HIV-1)**	MDM	M-Sec Signaling cascade	HIV	Hashimoto et al., 2016
	**HMPV P** **phosphoprotein**	Lung epithelial BEAS-2B cells	Stimulation of GTPases involved in actin polymerization	HMPV N protein HMPV particles	El Najjar et al., 2016
	**US3 protein kinase (Pseudorabies)**	Swine testicle (ST) cells; RK13 cells	E-cadherin β-catenin	GFP, mitochondria, virions-containing vesicles	Jansens et al., 2017
**Neurodegenerative disease**	**Infectious prions PrP^Sc^**	Neuronal CAD	Colocalization of PrPSc with EEA1 and Vamp3	Endosomes containing PrPSc cells	Zhu et al., 2015
	**α-synuclein (PD)**	Neurons	---	Lysosomal vesicles containing α-synuclein	Abounit et al., 2016a, 2016b
	**mHTT (HD)**	CAD neuronal cells	---	mHTT aggregates	Costanzo et al., 2013
	**Tau (AD)**	Neurons CAD cells	Tau co-localization with actin in TNTs	Tau aggregates	Tardivel et al., 2016

## Abbreviations

ACAP2	Arf GAP with coiled coil: ankyrin repeat and PH Domains 2
AD	Alzheimer’s disease
Akt	Protein kinase B/serine/threonine kinase
AML	Acute myeloid leukemia
ARF6	ADP-ribosylation factor 6
ATP	Adenosine triphosphate
BDNF	Brain-derived neurotrophic factor
BMP	Bone morphogenetic protein
BMSC	Bone marrow stromal cells
CJD	Creutzfeld-Jakob disease
CLU	Clusterin
CNS	Central nervous system
CNTF	Ciliary neurotrophic factor
Cx43	Connexin 43
EGFP	Enhanced green fluorescent protein
EGFR	Epidermal growth factor receptor
EHD1	EH domain containing 1
Eps8	Epidermal growth factor receptor pathway 8
ER	Endoplasmic reticulum
ERp29	Endoplasmic reticulum protein 29
EV	Extracellular vesicle
FGF2	Fibroblast growth factor 2
GABA	Gamma-aminobutyric acid
GAP-43	Growth associated protein 43
GAPDH	Glyceraldehyde 3-phosphate dehydrogenase
GBM	Glioblastoma
GDNF	Glial cell line-derived neurotrophic factor
GDP	Guanosine diphosphate
GFP	Green fluorescent protein
GGF2	Glial growth factor 2
GJ	Gap junctions
GSC	Glioblastoma stem cells
GTPase	Guanosine triphosphatase
H_2_O_2_	Hydrogen peroxide
HD	Huntington’s disease
HIV-1	Human immunodeficiency virus
HMPV	Human metapneumovirus
IGF-1	Insulin-like growth factor 1
LIF	Leukemia inhibitory factor
lncRNA	Long non-coding RNA
LPS	Lipopolysaccharide
LST-1	Leukocytic specific transcript-1
MDM	Monocytes derived macrophages
MGMT	O^6^-methylguanine-DNA methyltransferase
MHC	Major histocompatibility complex
mHTT	Mutant huntingtin
Miro1	Mitochondrial Rho GTPase 1
MSC	Mesenchymal stem cell
mtDNA	Mitochondrial desoxyribonucleic acid
mTOR	Mechanistic target of rapamycin
Myo10	Myosin 10
NGF	Nerve growth factor
NOX2	NADPH oxidase 2
NSG	Non obese diabetic (NOD)-scid gamma
PD	Parkinson’s disease
PDGF-AA	Platelet derived growth factor-AA
PENK	Proenkephalin
PI3K	Phosphoinositide 3 kinase
PrP^Sc^	Infectious prion
RalGPS2 ROS	Ras-independent guanine nucleotide exchange factor for RalA GTPase Reactive oxygen species
TGF-β	Transforming growth factor-β
TM	Tumor microtube
TMZ	Temozolomide
TNFα	Tumor necrosis factor α
TNT	Tunneling nanotube
Tthy1	Tweety homolog 1
VEGF	Vascular endothelial growth factor
WASP	Wiskott-Aldrich syndrome protein
WAVE	WASP-family verprolin-homologous protein
Wnt	Wingless and Int1
YB-1	Y-box binding protein-1
α-syn	α-synuclein
βCAMKII	Ca^2+^/Calmodulin-dependent protein kinase II
